# Bioelectrical Activity of Masticatory Muscles and Postural Stability Across TMD Subtypes

**DOI:** 10.3390/diagnostics16050799

**Published:** 2026-03-08

**Authors:** Aleksandra Dolina, Justyna Pałka, Magdalena Zawadka, Marcin Wójcicki, Monika Litko-Rola, Jacek Szkutnik, Piotr Gawda

**Affiliations:** 1 Department of Sports Medicine, Medical University of Lublin, Chodzki 15, 20-093 Lublin, Poland; magdalena.zawadka@umlub.edu.pl; 2 Interdisciplinary Scientific Group of Sports Medicine, Department of Sports Medicine, Medical University of Lublin, Chodzki 15, 20-093 Lublin, Poland; 3 Independent Unit of Functional Masticatory Disorders, Medical University of Lublin, Chodzki 6, 20-093 Lublin, Poland; marcin.wojcicki@umlub.edu.pl (M.W.); monika.litko.rola@umlub.edu.pl (M.L.-R.); jacek.szkutnik@umlub.edu.pl (J.S.)

**Keywords:** temporomandibular joint disorders, temporomandibular joint, electromyography, postural balance, posture, masticatory muscles, stomatognathic system

## Abstract

**Background**: Existing evidence suggests an association between temporomandibular disorders (TMDs) and alterations in body posture and balance; however, the mechanism underlying this relationship remains unknown. The present study aimed to investigate the associations between specific TMD subtypes, indices of bioelectrical activity of the masticatory muscles, and parameters of body posture and balance. **Methods**: The study followed a case–control study design. A total of 81 participants were enrolled, including 33 controls and 48 individuals with TMD, classified into myofascial (*n* = 14), articular (*n* = 17), and mixed (*n* = 17) subtypes. Diagnosis of temporomandibular disorders was carried out by prosthodontic specialists using the Polish adaptation of the Diagnostic Criteria for Temporomandibular Disorders. Masticatory muscle bioelectrical activity was assessed by surface electromyography. For statistical analysis, the Asymmetry Index and Functional Clenching Activity Indices were used. Static balance was evaluated with a pedobarographic platform. The sway area, velocity, and length of the Center of Pressure, as well as the foot contact area, were recorded and automatically calculated by the system. Measurements were performed under different mandibular conditions, with both eyes open and eyes closed. Correlation analyses were performed using Spearman Rank Order Correlation. Pearson’s Chi-squared test was used for the analysis of categorical variables. **Results**: Weak to moderate negative correlations were primarily observed, indicating that higher indices of masticatory muscle bioelectrical activity were associated with better postural balance, with distinct correlation patterns identified across different TMD subtypes. **Conclusions**: This exploratory study identified multiple correlations between masticatory muscle activity and postural or balance parameters, suggesting possible subtype-specific patterns in TMDs. However, the evidence remains preliminary and should be interpreted with caution, warranting further confirmatory and longitudinal research.

## 1. Introduction

Temporomandibular disorders (TMDs) represent an umbrella term encompassing a variety of dysfunctions of the stomatognathic system, characterized by heterogeneous symptoms, severity, and etiologies. These disorders involve the masticatory muscles, temporomandibular joints (TMJs), and associated structures, and constitute the most frequent non-dental cause of pain in the orofacial region [1,2,3]. The prevalence of TMDs is estimated to affect approximately 30% of the global population [4,5], with predictive models suggesting that by 2050, up to 44% of individuals may be affected [6]. Moreover, TMDs impose a substantial financial burden on healthcare systems [7]. Surface electromyography (sEMG) was first employed to investigate the activity of the masticatory muscles in 1949 [8]. Since then, it has become an increasingly valuable diagnostic and research tool in the field of TMDs, bruxism, dental treatment, and rehabilitation. Numerous studies have demonstrated that patients with TMDs exhibit altered patterns of masticatory muscle activity. Comparative analyses of the bioelectric activity of the masseter and temporalis muscles between symptomatic and asymptomatic individuals have revealed higher resting activity and lower functional activity in TMD patients [8,9]. Such neuromuscular changes may contribute to muscle fatigue and impaired function [10]. Differences in activity patterns have also been observed in relation to muscle asymmetry (right vs. left side) or functional activity indicators [11].

In recent years, increasing attention has been directed toward the potential associations between TMDs, posture and body balance; however, existing findings remain inconsistent [12,13,14,15,16,17,18,19,20,21,22,23,24,25,26,27]. While some studies have reported no meaningful relationship between TMD and postural alignment or balance, as well as their clinical implications [13,19,22], others have observed significant associations, emphasizing that not every type of this disorder may affect body posture, particularly global posture [12,17,18].

Researchers have emphasized the need to clarify the causal relationship between TMDs and postural disturbances, as well as to better understand the underlying mechanisms [12]. Several theoretical frameworks have been proposed to explain possible links between TMDs and postural control. Among them, the concepts of myofascial chains and tensegrity have received attention as potential biomechanical models. These models suggest that fascial continuity and neuromuscular interconnections may transmit tension or altered proprioceptive inputs across distant body regions, thereby influencing postural organization [28,29,30]. Previous studies have suggested that alterations in masticatory muscle bioelectrical activity may be functionally associated with changes in mandibular positioning and, consequently, with adaptations in head and cervical posture, which could secondarily affect plantar pressure distribution and balance through complex neuromuscular and biomechanical interconnections. Furthermore, unilateral chewing activity has been hypothesized to contribute to imbalances in cervical musculature and anterior myofascial pathways, which may affect postural alignment and stability [12]. Although these interpretations remain theoretical, they underscore the potential role of masticatory muscle function—and particularly the asymmetry of its bioelectrical activity—in the broader context of postural regulation. Some evidence suggests that such postural or balance disturbances are most frequently observed in individuals with myogenous forms of TMD, whereas articular subtypes appear to exert less influence on postural parameters [14]. However, both the existence of myofascial chains and their potential relevance for postural control remain hypothetical and require further empirical confirmation.

Considering the gaps in the current literature, and given that previous studies have not comprehensively examined the correlation between standardized surface electromyographic asymmetry indices of masticatory muscles and objective posturographic balance parameters across distinct TMD subtypes, further investigation is warranted. Therefore, the present study aimed to investigate the associations between different TMD subtypes, indices of bioelectrical activity of the masticatory muscles, and body posture and balance.

## 2. Materials and Methods

The study was carried out in compliance with the principles outlined in the Declaration of Helsinki and was approved by the Bioethics Committee of the Medical University of Lublin (KE-0254/256/12/2022). Prior to enrollment, all participants were thoroughly informed about the study protocol and provided written informed consent to participate. The participants were recruited from among students of the Medical University of Lublin and patients presenting for consultation at the temporomandibular disorders treatment clinic. The exclusion criteria were as follows:

- age outside 18–40 years;
- presence of spontaneous pain of the stomatognatic system;
- pain of the masticatory muscles or temporomandibular joint during posturographic examination;
- conditions potentially affecting body balance (e.g., neurological disorders);
- previous musculoskeletal injuries within the last 6 months;
- scoliosis or other evident postural abnormalities;
- visual disturbances if the last ophthalmological examination was conducted more than 12 months prior;
- presence of fewer than 28 teeth;
- previous craniofacial or head and neck injuries within the last 6 months;
- surgical treatment in the head and neck region within the last 6 months;
- open bite;
- crossbite;
- Angle’s Class II or III malocclusion;
- presence of orthodontic appliances;
- missing more than four teeth in both dental arches;
- lack of four support zones in the dental arches;
- possession of dental prostheses (regardless of type);
- professional athletes;
- sports training or conservative dental treatment within 24 h prior to examination;
- alcohol consumption within 24 h prior to examination;
- skin diseases in the head and neck region;
- neurological disorders in the head and neck region;
- neoplastic diseases regardless of type or location;
- body mass index (BMI) below 18.5 or above 24.99.

Static balance was assessed using the FreeMED MAXI pedobarographic platform (Sensor Medica, Guidonia Montecelio, Rome, Italy). Posturography is widely regarded as the gold standard for assessing both static and dynamic postural control under various conditions [31]. Sway area [mm^2^], velocity [mm/s] and length [mm] of the Center of Pressure (COP) and foot contact area [mm^2^] were recorded and automatically calculated by the system. Measurements were performed under three mandibular conditions: (1) resting position, (2) maximum voluntary clenching, and (3) maximum clenching on cotton rolls. Each trial lasted 30 s with 5 s intervals, conducted with eyes open (EO) and eyes closed (EC). Foot placement on the platform was standardized. Participants stood barefoot, facing a wall at 150 cm, arms relaxed alongside the body, and were instructed to maintain maximal stability in a quiet environment without focusing on any specific point [31].

**Figure 1:**
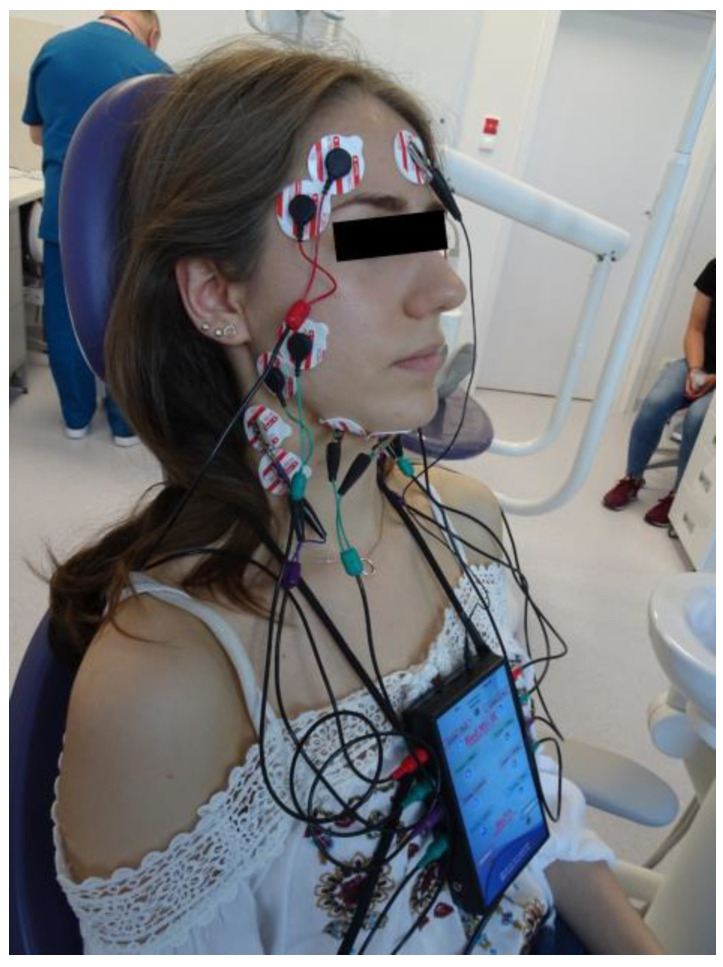
Electrode placement during surface electromyography examination.

Subsequently, the bioelectrical activity of the masticatory muscles was assessed using surface electromyography (sEMG). sEMG recordings were performed in the morning (9 a.m.–11 a.m.) to minimize daily fluctuations in muscle activity. Participants were seated in a dental chair with the head supported, torso perpendicular to the floor, and lower limbs extended and relaxed. Skin was cleansed with 90% ethyl alcohol prior to electrode placement. Ag/AgCl electrodes (30 mm diameter, 16 mm conductive surface; SORIMEX, Poland) were positioned on the anterior temporalis (TA), superficial masseter (MM), anterior bellies of the digastric muscle (DA), and sternocleidomastoid muscle (SCM) according to SENIAM guidelines [32] and Ferrario et al. [33], with a reference electrode on the forehead (Figure 1). Recordings were obtained using an 8-channel BioEMG III™ system with the BioPak Measurement System, version 8.8 (BioResearch Associates, Inc., Milwaukee, WI, USA). The sampling frequency was 2000 Hz, and the bandwidth was set between 30 and 1000 Hz. The manufacturer’s NoiseBuster function was used as an additional digital noise-reduction step, applied post-acquisition. This software filter provides a reduction in residual noise of approximately 40 dB, thereby enhancing signal quality prior to root-mean-square (RMS) processing and statistical analysis. Electrode–skin impedance was verified prior to each recording, with baseline noise < 5 µV RMS (corresponding to <5 kΩ) indicating acceptable signal quality. The recording protocol included three conditions: resting mandibular position (10 s), maximal voluntary clenching in the intercuspal position (three 3 s contractions, 2 s intervals; as hard as possible), and maximal voluntary clenching on dental rollers (three 3 s contractions, 2 s intervals; as hard as possible) [34,35]. For normalization, the mean root mean square (RMS) amplitude during each task was expressed as a percentage of the maximal RMS value obtained during MVC (%MVC). To ensure repeatability, all recordings were conducted by the same investigator under identical environmental conditions. Crosstalk was minimized by maintaining a minimum 20 mm interelectrode distance and precise electrode alignment along the muscle fibers [32].

Muscle asymmetry (MM, TA, DA, SCM) was quantified using an asymmetry index (AsI), calculated from root mean square (RMS) values:

*AsI* = (*RMS_right_* − *RMS_left_*)/(*RMS_right_* + *RMS_left_*) × 100

AsI values range from +100 (right muscle only) to −100 (left muscle only), with 0 indicating equal bilateral activity [36,37].

Functional Clenching Activity Indices (FCAI) [11] were applied to standardize the mean bioelectrical potentials:

Functional Clenching Symmetry Index for anterior temporalis (FCSITA) =  (FCITA-R − FCITA-L)/(FCITA-R + FCITA-L) × 100

Functional Clenching Symmetry Index for masseter (FCSIMM) =  (FCIMM-R − FCIMM-L)/(FCIMM-R + FCIMM-L) × 100

Functional Clenching Activity Index left-sided (FCAI-L) =  (FCIMM-L − FCITA-L)/(FCIMM-L + FCITA-L) × 100

Functional Clenching Activity Index right-sided (FCAI-R) =  (FCIMM-R − FCITA-R)/(FCIMM-R + FCITA-R) × 100

Functional Clenching Activity Index total (FCAI) =  (FCIMM − FCITA)/(FCIMM + FCITA) × 100

Posturographic and sEMG measurements were consistently performed by the same investigator (A.D.). Group allocation (study/control) was determined only after the completion of posturographic and sEMG assessments; therefore, the examiner remained blinded to participant allocation throughout data collection.

Diagnosis of temporomandibular disorders was conducted by prosthetics specialists (J.S.) according to the Polish version of the Diagnostic Criteria for Temporomandibular Disorders (DC/TMD) [38]. Based on the criteria, participants were allocated into a study group, comprising individuals with temporomandibular disorders, and a control group, consisting of participants without TMD symptoms. Next, the study group was subsequently categorized into disorder-specific subgroups:

- myofascial subtype (muscle pain) [39],
- articular subtype (disc disorders—disc displacement with/without reduction with/without limited opening; joint disorders—joint pain, degenerative joint disease) [39],
- and mixed subtype (combining myofascial and articular characteristics).

### Data Analysis

All statistical analyses were performed using the Statistica™ software package, version 14.00 (TIBCO Software Inc., Palo Alto, CA, USA). The normality of data distribution was assessed using the Shapiro–Wilk test. Group comparisons based on the type of disorder were conducted using the Kruskal–Wallis test, followed by post hoc Dunn’s test with Bonferroni correction. To estimate the effect size, eta squared (η^2^) was calculated as a measure of the proportion of variance in the ranked dependent variable explained by group membership. Interpretation of η^2^ follows general guidelines similar to those used for ANOVA-type effects: η^2^ ≈ 0.01 -small effect; η^2^ ≈ 0.06—medium effect; η^2^ ≥ 0.14—large effect. Correlation analyses were performed using Spearman Rank Order Correlation. The strength of the correlations was interpreted as follows: r = 0.10–0.39—weak correlation; r = 0.40–0.69—moderate correlation; r = 0.70–0.89—strong correlation; r = 0.90–1.00—very strong correlation [40]. Pearson’s Chi-squared test was used for the analysis of categorical variables. The level of statistical significance was set at α < 0.05. Descriptive statistics were presented as mean (M) and standard deviation (SD). Indicators related to the bioelectrical activity of muscles were presented using absolute values as the primary measure of asymmetry magnitude. Only statistically significant correlations are presented in the text. A full correlation analysis is included in the Supplementary Materials.

## 3. Results

### 3.1. Sample Size Calculation

An a priori power analysis was conducted using G*Power (version 3.1.9.7) for a one-way ANOVA: fixed effects. The analysis assumed a large effect size (f = 0.50), an alpha level of 0.05, and a desired statistical power of 0.80. The planned comparison included four independent groups. Based on these parameters, the required total sample size was calculated to be *n* = 48 participants, corresponding to 12 participants per group.

### 3.2. Characteristics of the Study Group

After applying the exclusion criteria, 81 participants remained eligible from the initial sample of 100 individuals. Among them, 33 participants were allocated to the control group, whereas 48 participants were assigned to the study group. Participants with TMDs were classified into subgroups according to the type of disorder: myofascial (*n* = 14), articular (*n* = 17), and mixed (*n* = 17).

The study groups did not differ significantly in terms of the number of females and males (χ^2^ = 2.02, *p* = 0.56). There were no statistically significant differences in age between the groups (H = 1.71, *p* = 0.64). The mean age ranged from 24.15 years (SD = 2.68) in the control group to 25.59 years (SD = 4.85) in the mixed group. Similarly, height did not differ significantly across groups (H = 3.60, *p* = 0.31), with mean values ranging from 169.00 cm (SD = 6.86; articular group) to 171.21 cm (SD = 6.46; control group).

Body weight was also comparable between groups (H = 1.05, *p* = 0.78), with mean values ranging from 61.35 kg (SD = 8.42; joint group) to 65.75 kg (SD = 17.05; muscular group). BMI values were similar as well (H = 2.43, *p* = 0.49), with the lowest mean observed in the joint group (21.42 kg/m^2^, SD = 2.04) and the highest in the muscular group (22.59 kg/m^2^, SD = 3.67).

### 3.3. Postural Stability

Kruskal–Wallis H tests indicated a number of statistically significant differences between the groups in terms of postural stability parameters (Table 1). Post hoc analysis revealed statistically significant differences in sway path length between the control group and the muscular group (*p* = 0.02) as well as the mixed group (*p* = 0.009) during jaw clenching with eyes open. Additionally, a significant difference was found between the control and mixed groups during jaw clenching with eyes closed (*p* = 0.02). Statistically significant differences were also observed when comparing the control group with the muscular group (eyes open: *p* = 0.01; eyes closed: *p* = 0.03), the mixed group (eyes open: *p* < 0.001; eyes closed: *p* = 0.004), and between the joint and mixed groups (eyes open: *p* = 0.03; eyes closed: *p* = 0.01) during jaw clenching on cotton rolls.

In terms of the ellipse area, a statistically significant difference was found between the control and muscular groups (*p* = 0.01) during jaw clenching with eyes open. No other significant differences between subgroups were observed for this parameter. The mean velocity during jaw clenching differed significantly between the control and mixed groups (eyes open: *p* = 0.02; eyes closed: *p* = 0.01), and between the control and muscular groups (eyes open: *p* = 0.001; eyes closed: *p* = 0.02). A statistically significant difference in mean velocity during jaw clenching on cotton rolls was also observed between the control and mixed groups (eyes open: *p* = 0.003; eyes closed: *p* = 0.001), and between the control and muscular groups (eyes open: *p* = 0.02; eyes closed: *p* = 0.002). The analysis of plantar contact area was presented in a previous study by the authors [24].

### 3.4. Electromyographic Indicators

When analyzing indices reflecting the electrical activity of the masticatory muscles, statistically significant differences between groups were found for only one parameter. The groups differed significantly in the AsI-TA index during clenching on cotton rolls (*p* = 0.003). Post hoc analysis revealed that the mixed group had significantly higher ASI-TA values compared to both the control group (*p* = 0.008) and the articular group (*p* = 0.002). Additionally, the myofascial group demonstrated significantly higher ASI-TA values than the articular group (*p* = 0.03). No statistically significant differences were observed between the control and articular or myofascial groups. Detailed results are presented in Table 2.

### 3.5. Correlation Analysis Results

For transparency and completeness, the entire set of correlation results, comprising both significant and non-significant associations, is presented in the Supplementary Materials (Tables S1–S4). Only statistically significant correlations are included in the main text to maintain clarity and prevent excessive data redundancy.

In the control group, the most frequent statistically significant negative correlations of weak to moderate strength were observed between the FCAI parameters and the plantar contact area. Both at rest and during teeth clenching, the contact area of the feet increased as the FCAI-R value decreased. Correlation strength ranges from weak (FCAIR-R & Foot contact area rest L: r = −0.37, *p* = 0.04) to moderate (FCAIR-R & Foot contact area rest R: r = −0.50, *p* = 0.003). Detailed results are presented in Table 3.

In the myofascial group, the most frequent statistically significant negative correlations of moderate strength were found between the AsI-DA index during teeth clenching and balance parameters: sway path length and mean velocity. An increase in the AsI-DA clenching index was associated with a decrease in sway path length and mean velocity. (EO and EC clenching on cotton rolls r = 0.60, *p* = 0.02; EC rest r = −0.57, *p* = 0.03; EC clenching r = −0.62, *p* = 0.02; EO clenching on cotton rolls r = −0.61, *p* = 0.02). Additionally, statistically significant positive correlations were found between the plantar contact area and the AsI-DA index during clenching on cotton rolls, indicating that greater AsI-DA values were associated with a higher contact area. (clenching L r = 0.54, *p* = 0.047; clenching R r = 0.58, *p* = 0.03; clenching on cotton rolls L r = 0.73, *p* = 0.005). Detailed correlation results are presented in Table 4.

In the mixed group, the highest number of statistically significant correlations was observed between balance parameters (sway path length, ellipse area, mean velocity) and the AsI-TA index during clenching on cotton rolls. These were negative correlations of moderate to strong strength (sway path length EO clenching on cotton rolls r = −0.51, *p* = 0.04; ellipse area EO rest r = −0.81, *p* < 0.001), indicating that higher AsI-TA clenching values were associated with lower balance parameter values. Furthermore, the ellipse area parameter showed a negative correlation with FCAI-TA (EC rest r = −0.49, *p* = 0.045; EC clenching on cotton rolls r = −0.72, *p* = 0.001), suggesting that higher FCAI-TA values were associated with lower ellipse area values. Detailed results are shown in Table 5.

In the articular group, statistically significant negative correlations of moderate strength were found between balance parameters (sway path length, ellipse area, mean velocity) and resting AsI values for the MM, SCM, and DA muscles. Correlation ranges from r = −0.48 (mean velocity EC clenching on cotton rolls & AsI-MM rest, *p* = 0.049) to r = −0.57 (ellipse area EO clenching & AsI-DA rest, *p* = 0.02). These correlations indicate that higher resting AsI values were associated with lower values of balance parameters. Detailed results are presented in Table 6.

## 4. Discussion

The present study aimed to analyze the relationships between specific subtypes of temporomandibular disorders, indices of masticatory muscle bioelectrical activity, and body posture and balance.

We primarily observed negative correlations of moderate strength. This indicated that higher indices of masticatory muscle bioelectrical activity were associated with better parameters of postural balance. Our initial hypothesis, however, assumed the opposite relationship. A more detailed analysis revealed that this inverse pattern was not confined to a single muscle or condition, but varied across TMD subtypes. In the myofascial group, moderate negative correlations were primarily observed between the AsI-DA index during clenching and balance parameters, indicating that greater digastric anterior asymmetry was associated with reduced sway path length and mean velocity. In the mixed group, the largest number of significant associations concerned the AsI-TA index during clenching on cotton rolls, where higher asymmetry corresponded to lower sway path length and ellipse area values. In the articular group, negative correlations were identified mainly between resting AsI values of the MM, SCM, and DA muscles and balance parameters, suggesting that greater resting asymmetry was associated with better postural stability. In contrast, in the control group, correlations were predominantly observed between FCAI parameters and plantar contact area rather than classical sway measures. Overall, these findings indicate that the relationship between masticatory muscle asymmetry and postural control is subtype-dependent and condition-specific rather than uniform across groups.

It is difficult to directly compare the present findings with other research, as thus far only one study has investigated the bioelectrical activity of the masticatory muscles in the context of global posture. Valentino et al. [41] suggested a functional association between the bioelectrical tension of the masseter and temporalis muscles and foot deformities such as valgus and flatfoot. Specifically, a congenital right valgus foot was associated with increased tension of the right masseter, whereas a valgus foot on the left side was linked to decreased resting tension of the left masseter. The authors explained this relationship by the presence of mechanoreceptors in the tendons of the foot, which transmit information about foot alignment via the spinocerebellar tract and medullary nuclei gracilis and cuneatus, followed by the medial lemniscus and thalamocortical tract to the sensory cortex. From there, an efferent motor response is directed toward synapses of neurons in the trigeminal motor nucleus, ultimately leading to activation of the masticatory muscles [41]. Nonetheless, these findings are difficult to extrapolate to our results, as Valentino et al. focused on asymptomatic individuals rather than patients with TMD.

In numerous other studies examining the association between TMDs and posture or balance, considerations of the underlying mechanisms remain largely hypothetical [15,16,18,19,20].

In a study by Di Paolo et al. [15] assessed patients with unilateral disc displacement with reduction compared to a control group. The TMDs group demonstrated alterations in thoracic kyphosis angle, lumbar lordosis, and lateral deviations of the spine. However, statistically significant differences between groups were observed only for lateral spinal deviation (*p* = 0.02), which the authors attributed to abnormal condyle–disc relationships. Within the TMDs group, correlations were noted between lateral and rotational spinal deviations and palpatory muscle pain. The authors explained these associations as potentially stemming from hyperactivity of the masticatory muscles (particularly the bilateral lateral pterygoids and the left masseter), although they emphasized that the direction of causality could not be established in their study [15].

Another study focusing on disc displacement without reduction and balance suggested no association between the artrogenic subtype of TMDs and postural control. In this group, no differences were found in mandibular rest position or maximal clenching on cotton rolls under either eyes-open or eyes-closed conditions, on stable or unstable surfaces. By contrast, in asymptomatic controls, clenching improved balance by reducing the center of pressure sway velocity [20]. In their discussion, the authors interpreted this finding as being mediated by feedback from periodontal pressure receptors that control jaw adductor muscles. These muscles, through their functional linkage to cervical musculature, may influence postural control. The authors suggested that masticatory and cervical muscles contract simultaneously during maximal clenching to enhance proprioception and improve balance [42], which would explain the reduction in postural sway in the control group. In our study, in the mixed TMD subgroup, the highest number of statistically significant correlations was observed between balance parameters (sway path length, ellipse area, mean velocity) and the AsI-TA index during clenching on cotton rolls. Clenching on cotton rolls is commonly used as a standardized experimental condition that reduces the influence of individual occlusal contacts and modifies afferent input from periodontal receptors [43]. It is therefore possible that this condition enhances the detectability of subtle associations between masticatory muscle asymmetry and postural regulation. However, the strength of the observed correlations was predominantly weak to moderate, and similar patterns were not consistently identified across all TMD subtypes. Consequently, this observation does not imply specific clinical relevance or support the use of cotton-roll clenching as a diagnostic or therapeutic tool. Rather, it may indicate that standardized occlusal conditions provide a sensitive experimental context for exploring interactions between the stomatognathic system and postural control, which requires further investigation in longitudinal and mechanistic studies.

Souza et al. [16] reported that TMDs participants exhibited multiple global postural deviations compared with asymptomatic individuals. The symptomatic group demonstrated greater cervical distance, right calcaneal valgus, reduced pelvic tilt, and increased rearfoot loading relative to controls. The authors explained the potential TMD–posture link by referencing interactions between the trigeminal system, neural structures responsible for neural control of posture, and myofascial chains. Consequently, tension within the stomatognathic system may impair neural control of the posture [16].

Nota et al. examined exclusively patients with myogenous TMDs and compared them with asymptomatic controls. Significant differences were observed in the center of pressure area and sway velocity under various mandibular conditions. For symptomatic patients, clenching was associated with deterioration of balance parameters [18]. These results align with our earlier findings, where the largest differences in sway area and velocity were observed between controls and patients with the myogenous subtype of TMDs [17]. Nota et al. attributed their observations to the presence of polysynaptic and oligosynaptic neural pathways linking the masticatory muscles and the vestibular labyrinth, which could explain why postural and balance impairments are primarily associated with the myogenous subtype of TMD. However, the authors emphasized that this explanation remains hypothetical [18].

Rocha et al. [19] found no significant differences in balance parameters or postural deviations between patients with unilateral, painless disc displacement and controls. They suggested that the TMD–posture relationship may depend not on the mere presence of TMD, but rather on the presence of pain during its course. Pain, therefore, might serve as the stimulus driving postural adaptation in TMD patients [19]. In our study, however, individuals with spontaneous stomatognathic pain or pain elicited during posturographic or sEMG examination were excluded. Consequently, pain adaptation cannot account for our findings. Nevertheless, in most prior studies, patients with painful TMDs were typically included, and thus it cannot be excluded that pain acted as an intermediary factor in the TMD–posture relationship in those investigations. Given that painful TMD is associated with altered bioelectrical activity of the masticatory muscles [44], future studies assessing the relationship between TMDs and postural or balance parameters should systematically monitor pain intensity as well as bioelectrical activity patterns of the masticatory and cervical muscles. Moreover, TMD frequently coexist with other pain conditions. Bonato et al. demonstrated that patients with TMDs had a 5.5-fold higher likelihood of pain in other body regions compared with controls, with the muscular subtype being associated with the greatest number of painful sites. The authors attributed these findings to the involvement of central sensitization mechanisms in the pathophysiology of TMDs [45]. Thus, future research on the transmission of TMD dysfunctions to distant body regions should also consider the potential role of central sensitization.

Although certain patterns can be discerned in our results, their overall ambiguity prevents any definitive conclusions. This inconsistency may, at least in part, be attributed to the absence of statistically significant differences in muscle asymmetry indices and functional activity ratios between the individual TMD subtypes. We initially hypothesized that distinct bioelectrical activity patterns across TMD subtypes might act as mediating factors in the relationship between TMD and postural balance. However, this assumption was not substantiated by the results obtained. Several plausible explanations for this outcome may be considered. Given the potential influence of pain on posture, patients reporting pain during postural or balance assessment or during masticatory muscle bioelectrical activity recording were excluded. While this exclusion was methodologically justified for assessing posture and balance, it may have confounded the bioelectrical muscle activity findings. According to the literature, abnormal bioelectrical activity patterns, including asymmetry, are more frequently observed in patients with painful TMDs [44]. Furthermore, as previously noted, it remains unclear whether TMD subtypes differ with respect to masticatory muscle bioelectrical activity [9]. Future investigations should therefore address various parameters of bioelectrical muscle activity without necessarily stratifying patients by TMD subtypes, while also incorporating pain as a variable potentially influencing adaptive postural and balance changes. The lack of statistical significance in a substantial proportion of tested associations may also be related to residual clinical heterogeneity within the defined TMD subtypes. Although participants were stratified according to DC/TMD criteria, patients within each subgroup may have differed in the severity and duration of dysfunction, the extent of functional adaptation, and the presence of coexisting conditions frequently associated with TMD, such as other pain complaints. Such variability may have attenuated group-level effects and reduced the detectability of consistent associations between bioelectrical muscle activity and postural parameters.

It should be noted that a large number of correlations were tested across different muscle indices, postural parameters, mandibular conditions, and TMD subtypes; only a subset of these associations reached statistical significance, while the majority were non-significant. Our findings are based on exploratory, cross-sectional analyses—characterized by the examination of multiple associations across diverse electromyographic and posturographic parameters without a single predefined primary outcome—and should therefore be interpreted with appropriate scientific caution and not overgeneralized beyond the observed data. It is also plausible that increasing the sample size within individual subgroups could enhance the consistency and robustness of the observed relationships. Importantly, the present findings should not be interpreted as supporting immediate changes in diagnostic algorithms or therapeutic decision-making in patients with temporomandibular disorders. The observed correlations do not establish causal relationships and do not justify the use of masticatory muscle bioelectrical activity or postural parameters as standalone diagnostic markers or treatment targets. At this stage, the results should be considered hypothesis-generating and intended to inform future mechanistic and longitudinal investigations rather than direct clinical application.

In summary, understanding the mechanisms underlying the transmission of stomatognathic dysfunctions to other body regions appears important for several reasons. First, given the multifactorial and complex etiology and the high prevalence of TMDs in the general population, it is essential to identify all risk factors that predispose to TMDs, contribute to its chronification, or exacerbate its clinical course. Second, elucidating the directionality of the relationship between TMDs and postural or balance deviations may enable clinicians to better identify the sources of patient pain and tailor targeted treatment strategies. Conversely, defining these associations could expand diagnostic protocols for patients with postural and balance disorders to include evaluation of the stomatognathic system. However, it should be noted that the present findings are exploratory and do not imply any immediate diagnostic or therapeutic implications. Further longitudinal studies are required to determine whether the observed associations may have clinical significance. Therefore, future studies should focus on the plausible mechanisms underlying these relationships and adopt a longitudinal design.

A limitation of our study is the large number of exclusion criteria applied. These criteria were designed to encompass factors typically controlled for in both posturographic and surface electromyography research. Furthermore, it was necessary to consider additional variables that may influence body posture—such as neurological or vestibular disorders—as well as those regarded in the literature as hypothetical contributors to postural balance and frequently associated with TMD, including occlusal disturbances. The authors acknowledge that the implementation of such comprehensive exclusion criteria may have affected the representativeness of the study sample. Nevertheless, rigorous control of potential confounding factors was considered methodologically more important than maximizing sample representativeness. An additional limitation of the present study concerns the relatively small sample size within specific TMD subgroups. Also, participants were recruited both from a TMD outpatient clinic and among university students. Although this recruitment strategy ensured an adequate sample size and reflected the typical demographic profile of TMD patients (advantage of women; age 20–40 years), it may still introduce spectrum bias and limit the generalizability of the findings. Future investigations using larger and more representative cohorts are warranted to strengthen the robustness and generalizability of these findings and to clarify the directionality of the relationship between TMD and postural disturbances, as current cross-sectional evidence does not permit causal inference. Another limitation of the study concerns the lack of randomization in the sequence of posturographic and electromyography tasks, as well as the short rest intervals between maximal clenching trials. These factors may have contributed to minor fatigue effects, which should be considered when interpreting the results. However, the sequence of posturographic tasks was determined by the technical configuration of the measurement system, which necessitated a fixed procedural order. Maintaining a standardized sequence across all participants reduced potential operator-dependent variability and minimized the risk of procedural errors. Furthermore, the duration and intensity of individual tasks were relatively short, which likely limited the magnitude of clinically meaningful fatigue effects.

## 5. Conclusions

The present study demonstrated that the relationships between masticatory muscle bioelectrical activity and postural or balance parameters differ between individuals with TMD and asymptomatic controls. Distinct patterns of association were observed across TMD subtypes, suggesting that bioelectrical activity of the masticatory muscles may interact with postural regulation in a subtype-dependent manner. However, the identified correlations were predominantly weak to moderate in strength and do not allow for causal or clinical conclusions.

## Figures and Tables

**Table 1:** Group comparisons in terms of static balance and posture parameters.

Variable	Control		Myofascial		Mixed		Articular		Statistics *p*-Value	n^2^
	M	SD	M	SD	M	SD	M	SD		
**Sway path length**										
EO rest	389.16	152.85	371.26	112.66	418.23	160.33	368.57	134.12	ns	−0.03
EC rest	423.86	182.59	392.57	129.03	433.25	175.40	350.86	102.25	ns	−0.02
EO clenching	340.01	130.40	469.74	142.77	498.37	186.08	368.69	123.60	0.003	0.15
EC clenching	369.02	151.90	473.79	150.36	512.46	183.42	369.48	118.59	0.005	0.13
EO clenching on cotton rolls	327.78	137.30	494.11	185.72	584.54	250.73	357.90	110.94	<0.001	0.25
EC clenching on cotton rolls	367.44	177.22	541.13	205.40	588.06	266.61	358.16	196.36	<0.001	0.20
**Ellipse area**										
EO rest	53.64	49.85	45.38	51.50	33.96	24.82	45.31	32.29	ns	−0.004
EC rest	44.44	40.34	42.22	49.29	46.38	40.91	61.69	67.02	ns	−0.02
EO clenching	28.52	27.11	73.59	69.47	57.24	54.10	33.42	22.48	0.01	0.11
EC clenching	33.54	37.62	50.81	48.78	60.97	55.44	55.02	72.70	ns	0.05
EO clenching on cotton rolls	30.21	25.95	75.48	79.28	45.49	38.67	38.31	29.77	ns	0.05
EC clenching on cotton rolls	36.54	34.15	53.82	56.63	55.11	54.51	58.77	71.37	ns	0.01
**Mean velocity**										
EO rest	12.52	4.75	13.54	5.29	14.12	5.29	12.46	4.52	ns	−0.02
EC rest	13.57	5.34	13.74	4.46	14.90	5.77	11.84	3.44	ns	−0.004
EO clenching	11.62	4.44	19.26	7.35	16.48	6.18	13.11	3.89	<0.001	0.19
EC clenching	12.85	5.28	19.16	7.17	18.31	6.67	12.87	3.76	0.001	0.16
EO clenching on cotton rolls	11.83	4.82	17.94	6.76	19.30	8.14	12.70	4.12	0.008	0.18
EC clenching on cotton rolls	13.38	6.16	18.27	7.21	21.20	8.59	11.89	4.48	<0.001	0.22

ns—not significant (*p* > 0.05); EO—eyes open; EC—eyes closed.

**Table 2:** Group comparisons in terms of EMG indicators.

Variable	Control		Myofascial		Mixed		Articular		Statistics *p*-Value	n^2^
	M	SD	M	SD	M	SD	M	SD		
**AsI**										
TA rest	20.24	15.68	15.35	11.59	17.60	11.32	16.19	11.76	ns	0.01
MM rest	21.07	16.68	16.86	14.98	12.32	10.80	16.35	14.94	ns	0.02
SCM rest	11.29	9.09	9.59	7.31	19.27	24.66	20.49	15.61	ns	0.02
DA rest	11.17	13.08	9.62	8.33	7.96	6.04	10.14	7.29	ns	0.03
TA clenching	13.06	12.23	19.16	11.38	16.28	14.12	10.72	10.91	ns	0.02
MM clenching	12.98	11.94	16.21	11.47	15.92	11.87	15.31	14.48	ns	0.00
SCM clenching	19.99	13.72	18.89	13.12	10.67	8.92	21.77	17.63	ns	0.011
DA clenching	20.86	15.43	22.26	14.41	19.31	9.41	20.06	15.03	ns	0.01
TA clenching on cotton rolls	10.91	8.62	17.82	9.79	22.74	14.64	8.56	7.80	0.003	0.06
MM clenching on cotton rolls	13.50	12.23	9.16	6.00	13.47	10.16	11.00	12.01	ns	0.04
SCM clenching on cotton rolls	15.45	12.62	19.45	11.90	13.15	10.41	18.12	14.49	ns	0.03
DA clenching on cotton rolls	18.60	15.62	20.02	12.80	14.26	9.40	20.52	13.99	ns	0.02
**FCAI**										
Right	27.61	24.79	24.25	21.16	32.05	25.44	26.97	21.15	ns	0.02
Left	25.08	20.54	30.79	15.44	35.41	17.80	19.31	16.49	ns	0.03
Total	22.64	20.62	20.54	17.01	32.13	22.01	21.67	16.87	ns	0.02
TA	24.24	19.80	21.56	13.09	21.35	17.90	14.43	10.17	ns	0.04
MM	23.21	19.02	21.84	12.97	19.90	14.66	20.75	15.50	ns	0.03

AsI—index of asymmetry; DA—anterior bellies of the digastric muscles; FCAI—Functional Clenching Activity Indices; MM—superficial masseter; ns—not significant (*p* > 0.05); SCM—sternocleidomastoid muscle; TA—anterior temporalis.

**Table 3:** Correlations of static balance and foot posture parameters and EMG indicators in the control group.

Variable	AsI-DA Rest	FCAI-R	FCAI-L	FCAI-Total
**Sway path length**				
EO rest	0.19	0.20	−0.06	−0.08
EC rest	0.33	−0.16	0.04	−0.22
EO clenching	0.16	0.05	−0.11	−0.20
EC clenching	0.28	−0.01	−0.22	−0.22
EO clenching on cotton rolls	0.15	0.11	−0.15	−0.22
EC clenching on cotton rolls	0.30	−0.04	−0.03	−0.22
**Ellipse area**				
EO rest	0.23	0.29	−0.14	0.07
EC rest	0.09	0.30	−0.06	0.19
EO clenching	0.23	0.23	−0.13	0.03
EC clenching	0.05	0.26	−0.18	−0.01
EO clenching on cotton rolls	0.12	0.16	−0.22	−0.05
EC clenching on cotton rolls	0.08	0.43 *	−0.06	0.05
**Mean velocity**				
EO rest	0.08	0.12	−0.18	−0.21
EC rest	0.42 *	−0.09	0.08	−0.10
EO clenching	0.23	−0.06	−0.15	−0.19
EC clenching	0.30	0.05	−0.22	−0.03
EO clenching on cotton rolls	0.12	−0.04	−0.14	−0.24
EC clenching on cotton rolls	0.18	−0.04	−0.22	−0.28
**Foot contact area**				
rest L	−0.19	−0.50 *	−0.32	−0.38 *
rest R	−0.15	−0.37 *	−0.12	−0.14
clenching L	−0.30	−0.43 *	−0.42 *	−0.32
clenching R	−0.17	−0.37 *	−0.11	−0.13
clenching on cotton rolls L	−0.13	−0.29	−0.14	−0.10
clenching on cotton rolls R	−0.12	−0.29	0.04	−0.10

*—statistically significant correlation; AsI—index of asymmetry; DA—anterior bellies of the digastric muscles; EC—eyes closed; EO—eyes open; FCAI-L—Functional Clenching Activity Indices left-sided; FCAI-R—Functional Clenching Activity Indices right-sided; FCAI-total—Functional Clenching Activity Indices total; L—left side; R—right side.

**Table 4:** Correlations of static balance and foot posture parameters and EMG indicators in the myofascial group.

Variable	AsI-DA Clenching	AsI-MM Clenching on Cotton Rolls	AsI-DA Clenching on Cotton Rolls	FCAI-R	FCAI-Total	FCAI-MM
**Sway path length**						
EO rest	−0.33	0.44	−0.21	−0.20	−0.03	0.05
EC rest	−0.50	0.04	−0.22	−0.07	−0.25	0.13
EO clenching	−0.27	0.43	0.00	−0.23	−0.19	0.21
EC clenching	−0.46	0.28	−0.10	−0.26	−0.22	0.11
EO clenching on cotton rolls	−0.60 *	0.34	−0.33	−0.31	−0.04	−0.05
EC clenching on cotton rolls	−0.60 *	0.31	−0.22	−0.32	−0.03	−0.11
**Ellipse area**						
EO rest	−0.10	−0.15	0.04	−0.52	−0.32	0.37
EC rest	0.20	−0.03	0.19	−0.38	−0.57 *	0.39
EO clenching	−0.16	0.32	0.21	−0.42	−0.34	0.53 *
EC clenching	−0.02	0.24	0.06	−0.44	−0.16	0.31
EO clenching on cotton rolls	0.06	0.24	0.16	−0.75 *	−0.34	0.15
EC clenching on cotton rolls	0.20	−0.21	0.09	−0.28	−0.20	0.45
**Mean velocity**						
EO rest	−0.36	0.60 *	−0.13	−0.34	−0.08	0.10
EC rest	−0.57 *	0.01	−0.28	−0.05	−0.14	0.09
EO clenching	−0.40	0.45	−0.16	−0.09	0.13	0.02
EC clenching	−0.62 *	0.19	−0.46	0.05	0.27	−0.16
EO clenching on cotton rolls	−0.61 *	0.31	−0.31	−0.29	0.04	−0.01
EC clenching on cotton rolls	−0.47	0.32	−0.26	−0.27	−0.01	−0.02
**Foot contact area**						
rest L	0.32	0.27	0.50	−0.05	−0.10	0.28
rest R	0.17	0.31	0.50	−0.01	−0.08	0.27
clenching L	0.43	0.13	0.54 *	0.13	0.02	0.10
clenching R	0.20	0.30	0.58 *	0.03	−0.11	0.36
clenching on cotton rolls L	0.52	0.38	0.73 *	−0.10	−0.18	0.25
clenching on cotton rolls R	0.30	0.48	0.55	−0.01	−0.02	0.35

*—statistically significant correlation; DA—anterior bellies of the digastric muscles; EC—eyes closed; EO—eyes open; FCAI-R—Functional Clenching Activity Indices right-sided; FCAI-total—Functional Clenching Activity Indices total; FCAI-MM—Functional Clenching Activity Indices of masseter; L—left side; MM—superficial masseter; R—right side.

**Table 5:** Correlations of static balance and foot posture parameters and EMG indicators in the mixed group.

Variable	AsI-SCM Rest	AsI-TA Clenching on Cotton Rolls	FCAI-L	FCAI-TA	FCAI-MM
**Sway path length**					
EO rest	−0.30	−0.48	−0.43	0.07	−0.22
EC rest	−0.19	−0.47	−0.38	0.00	−0.19
EO clenching	−0.38	−0.56 *	−0.35	0.09	−0.36
EC clenching	−0.26	−0.51 *	−0.27	0.17	−0.26
EO clenching on cotton rolls	−0.33	−0.51 *	−0.50 *	−0.03	−0.24
EC clenching on cotton rolls	−0.13	−0.44	−0.12	0.09	−0.15
**Ellipse area**					
EO rest	−0.49	−0.81 *	−0.51 *	−0.52 *	−0.51 *
EC rest	−0.47	−0.37	−0.44	−0.49 *	−0.10
EO clenching	−0.47	−0.71 *	−0.34	−0.21	−0.49 *
EC clenching	−0.33	−0.57 *	−0.35	−0.67 *	−0.09
EO clenching on cotton rolls	−0.55 *	−0.75 *	−0.49 *	−0.51 *	−0.47
EC clenching on cotton rolls	−0.41	−0.60 *	−0.49 *	−0.72 *	−0.25
**Mean velocity**					
EO rest	−0.37	−0.39	−0.39	0.14	−0.24
EC rest	−0.29	−0.47	−0.42	−0.02	−0.28
EO clenching	−0.37	−0.53 *	−0.31	0.11	−0.39
EC clenching	−0.36	−0.63 *	−0.49 *	−0.01	−0.38
EO clenching on cotton rolls	−0.26	−0.54 *	−0.39	−0.10	−0.27
EC clenching on cotton rolls	−0.24	−0.54 *	−0.16	−0.02	−0.21
**Foot contact area**					
rest L	0.28	0.35	0.50 *	0.07	−0.16
rest R	0.30	0.31	0.30	0.06	−0.06
clenching L	0.18	0.31	0.31	0.09	−0.28
clenching R	0.17	0.34	0.26	0.10	−0.18
clenching on cotton rolls L	0.18	0.39	0.43	0.09	−0.22
clenching on cotton rolls R	0.39	0.41	0.38	0.26	−0.16

*—statistically significant correlation; AsI—index of asymmetry; EC—eyes closed; EO—eyes open; FCAI-L—Functional Clenching Activity Indices left-sided; FCAI-MM—Functional Clenching Activity Indices of masseter; FCAI-TA—Functional Clenching Activity Indices of temporalis anterior; L—left side; R—right side; SCM—sternocleidomastoid muscle; TA—anterior temporalis.

**Table 6:** Correlations of static balance and foot posture parameters and EMG indicators in the articular group.

Variable	AsI-MM Rest	AsI-SCM Rest	AsI-DA Rest
**Sway path length**			
EO rest	−0.28	0.39	−0.01
EC rest	−0.35	0.06	0.11
EO clenching	−0.36	0.22	0.06
EC clenching	−0.30	0.17	0.06
EO clenching on cotton rolls	−0.32	0.27	−0.01
EC clenching on cotton rolls	−0.52 *	0.14	0.12
**Ellipse area**			
EO rest	0.25	−0.43	−0.36
EC rest	0.15	−0.21	−0.48
EO clenching	0.32	−0.49 *	−0.57 *
EC clenching	0.19	−0.23	−0.50 *
EO clenching on cotton rolls	0.30	−0.35	−0.41
EC clenching on cotton rolls	−0.08	−0.26	−0.23
**Mean velocity**			
EO rest	−0.28	0.39	−0.01
EC rest	−0.37	0.09	0.14
EO clenching	−0.53 *	0.13	−0.07
EC clenching	−0.37	0.14	0.05
EO clenching on cotton rolls	−0.27	0.21	−0.18
EC clenching on cotton rolls	−0.48 *	0.38	0.15
**Foot contact area**			
rest L	−0.14	−0.01	0.28
rest R	−0.21	0.11	0.21
clenching L	−0.11	0.04	0.20
clenching R	−0.10	0.01	0.12
clenching on cotton rolls L	0.09	−0.12	0.02
clenching on cotton rolls R	−0.01	−0.14	0.04

*—statistically significant correlation; AsI—index of asymmetry; DA—anterior bellies of the digastric muscles; EC—eyes closed; EO—eyes open; L—left side; MM—masseter; R—right side; SCM—sternocleidomastoid muscle.

## Data Availability

The data supporting the findings of this study are available from the corresponding author upon request.

## Supplementary Materials

The following supporting information can be downloaded at: https://www.mdpi.com/article/10.3390/diagnostics16050799/s1, Table S1: Correlations of static balance and posture parameters and EMG indicators in the control group. Table S2: Correlations of static balance and posture parameters and EMG indicators in the myofacial group. Table S3: Correlations of static balance and posture parameters and EMG indicators in the mixed group. Table S4: Correlations of static balance and posture parameters and EMG indicators in the articular group. Tables S1–S4 provide the complete results of the correlation analyses between masticatory muscle bioelectrical activity indices and parameters of postural balance and foot posture.

## Abbreviations

The following abbreviations are used in this manuscript:

AsI	Asymmetry Index
BMI	Body mass index
COP	Center of Pressure
DA	Anterior bellies of the digastric muscle
DC/TMD	Diagnostic Criteria for Temporomandibular Disorders
EO	Eyes Open
EC	Eyes Closed
FCAI	Functional Clenching Activity Indices
MM	Masseter muscle
RMS	Root mean square
SCM	Sternocleidomastoid muscle
sEMG	Surface electromyography
TA	Temporalis anterior
TMDs	Temporomandibular Disorders

## References

[1] Orzeszek S., Waliszewska-Prosol M., Ettlin D., Seweryn P., Straburzynski M., Martelletti P., Jenca A., Wieckiewicz M. (2023). Efficiency of Occlusal Splint Therapy on Orofacial Muscle Pain Reduction: A Systematic Review. BMC Oral Health.

[2] Li D.T.S., Leung Y.Y. (2021). Temporomandibular Disorders: Current Concepts and Controversies in Diagnosis and Management. Diagnostics.

[3] Zieliński G., Pająk-Zielińska B. (2024). Association between Estrogen Levels and Temporomandibular Disorders: An Updated Systematic Review. Int. J. Mol. Sci..

[4] Valesan L.F., Da-Cas C.D., Réus J.C., Denardin A.C.S., Garanhani R.R., Bonotto D., Januzzi E., de Souza B.D.M. (2021). Prevalence of Temporomandibular Joint Disorders: A Systematic Review and Meta-Analysis. Clin. Oral Investig..

[5] Zieliński G., Pająk-Zielińska B., Ginszt M. (2024). A Meta-Analysis of the Global Prevalence of Temporomandibular Disorders. J. Clin. Med..

[6] Zieliński G. (2025). Quo Vadis Temporomandibular Disorders? By 2050, the Global Prevalence of TMD May Approach 44%. J. Clin. Med..

[7] Slade G.D., Ohrbach R., Greenspan J.D., Fillingim R.B., Bair E., Sanders A.E., Dubner R., Diatchenko L., Meloto C.B., Smith S. (2016). Painful Temporomandibular Disorder: Decade of Discovery from OPPERA Studies. J. Dent. Res..

[8] Zieliński G., Gawda P. (2024). Surface Electromyography in Dentistry—Past, Present and Future. J. Clin. Med..

[9] Dinsdale A., Liang Z., Thomas L., Treleaven J. (2021). Is Jaw Muscle Activity Impaired in Adults with Persistent Temporomandibular Disorders? A Systematic Review and Meta-Analysis. J. Oral Rehabil..

[10] Ries L.G.K., Graciosa M.D., Soares L.P., Sperandio F.F., Santos G.M., Degan V.V., Gadotti I.C. (2016). Effect of Time of Contraction and Rest on the Masseter and Anterior Temporal Muscles Activity in Subjects with Temporomandibular Disorder. CoDAS.

[11] Ginszt M., Zieliński G. (2021). Novel Functional Indices of Masticatory Muscle Activity. J. Clin. Med..

[12] Minervini G., Franco R., Marrapodi M.M., Crimi S., Badnjević A., Cervino G., Bianchi A., Cicciù M. (2023). Correlation between Temporomandibular Disorders (TMD) and Posture Evaluated Trough the Diagnostic Criteria for Temporomandibular Disorders (DC/TMD): A Systematic Review with Meta-Analysis. J. Clin. Med..

[13] Manfredini D., Castroflorio T., Perinetti G., Guarda-Nardini L. (2012). Dental Occlusion, Body Posture and Temporomandibular Disorders: Where We Are Now and Where We Are Heading for. J. Oral Rehabil..

[14] Chaves T.C., Turci A.M., Pinheiro C.F., Sousa L.M., Grossi D.B. (2014). Static Body Postural Misalignment in Individuals with Temporomandibular Disorders: A Systematic Review. Braz. J. Phys. Ther..

[15] Di Paolo C., Papi P., Falisi G., Pompa G., Santilli V., Polimeni A., Fiorini A. (2020). Subjects with Temporomandibular Joint Disc Displacement and Body Posture Assessment via Rasterstereography: A Pilot Case-Control Study. Eur. Rev. Med. Pharmacol. Sci..

[16] Souza J.A., Pasinato F., Corrêa E.C.R., da Silva A.M.T. (2014). Global Body Posture and Plantar Pressure Distribution in Individuals with and without Temporomandibular Disorder: A Preliminary Study. J. Manip. Physiol. Ther..

[17] Dolina A., Baszczowski M., Zawadka M., Sobiech L., Szkutnik J., Gawda P. (2025). The Impact of Various Subtypes of Temporomandibular Disorders on Body Balance—Preliminary Study. Gait Posture.

[18] Nota A., Tecco S., Ehsani S., Padulo J., Baldini A. (2017). Postural Stability in Subjects with Temporomandibular Disorders and Healthy Controls: A Comparative Assessment. J. Electromyogr. Kinesiol..

[19] Rocha T., Castro M.A., Guarda-Nardini L., Manfredini D. (2017). Subjects with Temporomandibular Joint Disc Displacement Do Not Feature Any Peculiar Changes in Body Posture. J. Oral Rehabil..

[20] Zhang L., Xu L., Lu J., Cai B., Fan S. (2022). Static Balance in Participants with Temporomandibular Joint Disc Displacement without Reduction Versus Healthy Participants: A Cross-Sectional Study. Med. Sci. Monit. Int. Med. J. Exp. Clin. Res..

[21] Garstka A.A., Brzózka M., Bitenc-Jasiejko A., Ardan R., Gronwald H., Skomro P., Lietz-Kijak D. (2022). Cause-Effect Relationships between Painful TMD and Postural and Functional Changes in the Musculoskeletal System: A Preliminary Report. Pain Res. Manag..

[22] Manfredini D., Bender S.S., Häggman-Henrikson B., Durham J., Greene C.S. (2025). Temporomandibular Disorders: A New List of Key Points to Summarize the Standard of Care. Jpn. Dent. Sci. Rev..

[23] Almășan O., Kui A., Duncea I., Manea A., Buduru S. (2022). Temporomandibular Joint Disk Displacements in Class II Malocclusion and Cervical Spine Alterations: Systematic Review and Report of a Hypodivergent Case with MRI Bone and Soft Tissue Changes. Life.

[24] Dolina A., Pałka J., Zawadka M., Sobiech L., Baszczowski M., Szkutnik J. (2025). Comparison of Foot Contact Area and Plantar Pressures Distribution in Subject with Temporomandibular Disorders and Symptomatic Individuals. Wiad. Lek..

[25] Shetty S.K., Katageri A.I., Shetty R., Ragher M., Prabhu U.M. (2025). Evaluation of the Effect of Temporomandibular Joint Disorders on Craniocervical Posture: A Cross-Sectional Study. J. Indian Prosthodont. Soc..

[26] Gao D., Zhang S., Kan H., Zhang Q. (2024). Relationship between Cervical Angle and Temporomandibular Disorders in Young and Middle-Aged Population. CRANIO^®^.

[27] Antczak K., Pluta W., Lubkowski M., Radecka A., Lubkowska A. (2025). The Role of Posturography in the Diagnosis of Temporomandibular Disorders and Their Impact on Body Posture. Biomedicines.

[28] Cuccia A., Caradonna C. (2009). The Relationship Between the Stomatognathic System and Body Posture. Clinics.

[29] Cortese S., Mondello A., Galarza R., Biondi A. (2017). Postural Alterations as a Risk Factor for Temporomandibular Disorders. Acta Odontol. Latinoam..

[30] Wilke J., Krause F., Vogt L., Banzer W. (2016). What Is Evidence-Based About Myofascial Chains: A Systematic Review. Arch. Phys. Med. Rehabil..

[31] Baldini A., Nota A., Assi V., Ballanti F., Cozza P. (2013). Intersession Reliability of a Posturo-Stabilometric Test, Using a Force Platform. J. Electromyogr. Kinesiol..

[32] Hermens H.J., Freriks B., Disselhorst-Klug C., Rau G. (2000). Development of Recommendations for SEMG Sensors and Sensor Placement Procedures. J. Electromyogr. Kinesiol..

[33] Ferrario V.F., Sforza C., Miani A., D’Addona A., Barbini E. (1993). Electromyographic Activity of Human Masticatory Muscles in Normal Young People. Statistical Evaluation of Reference Values for Clinical Applications. J. Oral Rehabil..

[34] Wieczorek A., Loster J., Loster B.W. (2013). Relationship between Occlusal Force Distribution and the Activity of Masseter and Anterior Temporalis Muscles in Asymptomatic Young Adults. BioMed Res. Int..

[35] De Felício C.M., Sidequersky F.V., Tartaglia G.M., Sforza C. (2009). Electromyographic Standardized Indices in Healthy Brazilian Young Adults and Data Reproducibility. J. Oral Rehabil..

[36] Naeije M., McCarroll R.S., Weijs W.A. (1989). Electromyographic Activity of the Human Masticatory Muscles during Submaximal Clenching in the Inter-Cuspal Position. J. Oral Rehabil..

[37] Wieczorek A., Loster J.E. (2015). Activity of the Masticatory Muscles and Occlusal Contacts in Young Adults with and without Orthodontic Treatment. BMC Oral Health.

[38] Osiewicz M., Ciapała B., Bolt K., Kołodziej P., Więckiewicz M., Ohrbach R. (2024). Diagnostic Criteria for Temporomandibular Disorders (DC/TMD): Polish Assessment Instruments. Dent. Med. Probl..

[39] Schiffman E., Ohrbach R., Truelove E., Look J., Anderson G., Goulet J.-P., List T., Svensson P., Gonzalez Y., Lobbezoo F. (2014). Diagnostic Criteria for Temporomandibular Disorders (DC/TMD) for Clinical and Research Applications: Recommendations of the International RDC/TMD Consortium Network and Orofacial Pain Special Interest Group. J. Oral Facial Pain Headache.

[40] Luostarinen M., Remes A.M., Urpilainen P., Takala S., Venojärvi M. (2023). Correlation of Fatigue with Disability and Accelerometer-Measured Daily Physical Activity in Patients with Relapsing-Remitting MS. Mult. Scler. Relat. Disord..

[41] Valentino B., Melito F., Aldi B., Valentino T. (2002). Correlation between Interdental Occlusal Plane and Plantar Arches. An EMG Study. Bull. Group. Int. Rech. Sci. Stomatol. Odontol..

[42] Giannakopoulos N.N., Hellmann D., Schmitter M., Krüger B., Hauser T., Schindler H.J. (2013). Neuromuscular Interaction of Jaw and Neck Muscles during Jaw Clenching. J. Orofac. Pain.

[43] Ferrario V.F., Sforza C., Colombo A., Ciusa V. (2000). An Electromyographic Investigation of Masticatory Muscles Symmetry in Normo-Occlusion Subjects. J. Oral Rehabil..

[44] Szyszka-Sommerfeld L., Sycińska-Dziarnowska M., Spagnuolo G., Woźniak K. (2023). Surface Electromyography in the Assessment of Masticatory Muscle Activity in Patients with Pain-Related Temporomandibular Disorders: A Systematic Review. Front. Neurol..

[45] Bonato L.L., Quinelato V., De Felipe Cordeiro P.C., De Sousa E.B., Tesch R., Casado P.L. (2017). Association between Temporomandibular Disorders and Pain in Other Regions of the Body. J. Oral Rehabil..

