# Supplementary material for: Bioelectrical Activity of Masticatory Muscles and Postural Stability Across TMD Subtypes
Source: Diagnostics (Basel). 2026 Mar 8;16(5):799. doi: 10.3390/diagnostics16050799 (PMC12984159; doi:10.3390/diagnostics16050799)
Supplement: Supplementary file 1 [file diagnostics-16-00799-s001.zip › diagnostics-4117238-supplementary.pdf]

Bioelectrical Activity of Masticatory Muscles and Postural Stability Across TMD Subtypes

Aleksandra Dolina [1,*], Justyna Pałka [2], Magdalena Zawadka [1], Marcin Wójcicki [3], Monika Litko-Rola [3], Jacek Szkutnik [3] and Piotr Gawda [1,*]

[1] Department of Sports Medicine, Medical University of Lublin, Chodzki 15, 20-093 Lublin, Poland; magdalena.zawadka@umlub.edu.pl
[2] Interdisciplinary Scientific Group of Sports Medicine, Department of Sports Medicine, Medical University of Lublin, Chodzki 15, 20-093 Lublin, Poland
[3] Independent Unit of Functional Masticatory Disorders, Medical University of Lublin, Chodzki 6, 20-093 Lublin, Poland; marcin.wojcicki@umlub.edu.pl (M.W.); monika.litko.rola@umlub.edu.pl (M.L.-R.); jacek.szkutnik@umlub.edu.pl (J.S.)
* Correspondence: aleksandra.dolina.um@gmail.com (A.D.); piotr.gawda@umlub.edu.pl (P.G.); Tel.: +48-81448-6760 (A.D. & P.G.)

Table S1. Correlations of static balance and posture parameters and EMG indicators in the control group.

Variable	Rest				Clenching				Clenching on cotton rolls				FCAI				
	AsI - TA	AsI - MM	AsI - SCM	AsI - DA	AsI - TA	AsI - MM	AsI - SCM	AsI - DA	AsI - TA	AsI - MM	AsI - SCM	AsI - DA	R	L	total	TA	MM
**Sway path length**																	
EO rest	-0,14	0,34	-0,06	0,19	0,29	0,14	-0,03	-0,23	0,22	-0,14	-0,13	0,08	0,20	-0,06	-0,08	0,07	0,12
EC rest	-0,01	0,13	-0,14	0,33	0,13	-0,09	-0,02	-0,16	0,06	-0,06	-0,08	-0,03	-0,16	0,04	-0,22	0,18	-0,11
EO clenching	-0,22	0,24	-0,11	0,16	0,19	0,10	-0,07	-0,11	0,12	-0,12	-0,16	0,02	0,05	-0,11	-0,20	0,00	-0,03
EC clenching	-0,09	0,24	0,04	0,28	0,05	-0,04	0,14	-0,11	-0,01	-0,08	0,08	0,05	-0,01	-0,22	-0,22	0,04	0,03
EO clenching on cotton rolls	-0,14	0,20	-0,23	0,15	-0,03	-0,10	-0,07	0,02	0,08	-0,15	-0,14	0,17	0,11	-0,15	-0,22	-0,05	-0,04
EC clenching on cotton rolls	-0,02	0,04	-0,18	0,30	-0,02	-0,08	0,17	-0,03	0,18	-0,03	0,06	0,12	-0,04	-0,03	-0,22	0,08	0,03
**Ellipse area**																	
EO rest	0,10	0,11	-0,20	0,23	-0,02	0,16	0,09	-0,08	-0,10	0,05	0,03	0,14	0,29	-0,14	0,07	-0,05	0,01
EC rest	0,04	0,10	-0,18	0,09	-0,08	0,25	0,02	-0,16	-0,08	0,03	0,11	0,28	0,30	-0,06	0,19	-0,01	0,13
EO clenching	0,01	0,22	-0,10	0,23	-0,01	0,23	0,01	-0,23	-0,06	0,00	0,00	0,16	0,23	-0,13	0,03	-0,07	0,14
EC clenching	0,12	0,05	0,01	0,05	-0,12	0,11	0,07	-0,23	-0,23	-0,11	0,13	0,16	0,26	-0,18	-0,01	0,05	0,06
EO clenching on cotton rolls	0,05	0,19	-0,22	0,12	0,10	0,05	-0,01	-0,18	-0,02	-0,10	-0,09	0,08	0,16	-0,22	-0,05	0,02	-0,05
EC clenching on cotton rolls	0,12	0,26	0,05	0,08	0,01	0,28	0,04	-0,04	-0,09	-0,01	-0,01	0,34	0,43	-0,06	0,05	0,06	0,20
**Mean velocity**																	
EO rest	-0,23	0,18	-0,21	0,08	0,14	0,00	-0,01	-0,12	0,23	-0,17	-0,18	0,09	0,12	-0,18	-0,21	-0,12	-0,04
EC rest	0,00	0,16	-0,15	0,42	0,17	-0,01	0,06	-0,07	0,09	-0,01	-0,01	0,10	-0,09	0,08	-0,10	0,18	-0,04
EO clenching	-0,26	0,12	-0,14	0,23	0,13	0,03	-0,08	0,01	0,01	-0,08	-0,17	0,04	-0,06	-0,15	-0,19	-0,06	-0,18
EC clenching	-0,20	0,16	0,11	0,30	-0,05	0,03	0,27	-0,18	-0,10	-0,05	0,24	0,13	0,05	-0,22	-0,03	-0,07	0,04
EO clenching on cotton rolls	-0,20	0,02	-0,23	0,12	0,02	-0,21	0,00	0,18	0,13	-0,16	-0,10	0,12	-0,04	-0,14	-0,24	-0,07	-0,22
EC clenching on cotton rolls	-0,21	0,02	-0,14	0,18	-0,12	-0,15	0,15	0,06	0,02	-0,22	0,09	0,13	-0,04	-0,22	-0,28	-0,11	-0,10
**Foot contact area**																	
rest L	-0,13	-0,27	-0,21	-0,19	-0,15	0,04	-0,09	-0,21	0,13	-0,02	0,01	-0,18	-0,50	-0,32	-0,38	-0,12	-0,15
rest R	-0,12	-0,30	0,02	-0,15	-0,04	0,15	0,21	-0,13	0,25	0,13	0,25	-0,19	-0,37	-0,12	-0,14	-0,13	-0,12
clenching L	0,01	-0,19	-0,13	-0,30	-0,08	0,05	-0,11	-0,32	0,05	-0,08	-0,04	-0,20	-0,43	-0,42	-0,32	0,02	-0,07
clenching R	-0,13	-0,32	0,09	-0,17	-0,04	0,17	0,27	-0,13	0,21	0,14	0,29	-0,16	-0,37	-0,11	-0,13	-0,16	-0,10
clenching on cotton rolls L	0,09	-0,08	-0,24	-0,13	0,04	0,08	-0,11	-0,20	0,19	0,11	-0,03	-0,12	-0,29	-0,14	-0,10	0,17	-0,01
clenching on cotton rolls R	-0,14	-0,10	0,11	-0,12	-0,02	0,07	0,	-0,17	0,18	0,12	0,21	-0,14	-0,29	0,04	-0,10	-0,03	-0,01

AsI-index of asymmetry; DA- anterior bellies of the digastric muscles; EC – eyes closed; EO – eyes open; FCAI - Functional Clenching Activity Indice; L – left side; MM – masseter muscle; R – right side; SMC – sternocleidomastoid muscle; TA – temporalis anterior

Table S2. Correlations of static balance and posture parameters and EMG indicators in the myofacial group.

Variable	Rest				Clenching				Clenching on cotton rolls				FCAI				
	AsI - TA	AsI - MM	AsI - SCM	AsI - DA	AsI - TA	AsI - MM	AsI - SCM	AsI - DA	AsI - TA	AsI - MM	AsI - SCM	AsI - DA	R	L	total	TA	MM
**Sway path length**																	
EO rest	-0,01	0,08	0,16	-0,46	0,37	0,22	0,26	-0,33	0,00	0,44	0,20	-0,21	-0,20	0,10	-0,03	-0,11	0,05
EC rest	0,04	-0,06	-0,02	-0,24	0,04	-0,16	0,05	-0,50	-0,29	0,04	0,26	-0,22	-0,07	0,26	-0,25	-0,18	0,13
EO clenching	-0,02	0,14	0,26	-0,42	0,42	0,16	0,06	-0,27	0,08	0,43	0,19	0,00	-0,23	0,29	-0,19	-0,09	0,21
EC clenching	-0,01	-0,01	0,02	-0,20	0,23	0,00	0,11	-0,46	-0,18	0,28	0,28	-0,10	-0,26	0,27	-0,22	-0,25	0,11
EO clenching on cotton rolls	-0,02	-0,02	0,06	-0,27	0,30	0,11	0,32	-0,60	-0,34	0,34	0,39	-0,33	-0,31	0,05	-0,04	-0,25	-0,05
EC clenching on cotton rolls	0,01	-0,06	0,16	-0,12	0,31	0,09	0,27	-0,60	-0,35	0,31	0,45	-0,22	-0,32	0,09	-0,03	-0,24	-0,11
**Ellipse area**																	
EO rest	-0,16	0,10	-0,12	-0,11	-0,35	-0,18	-0,12	-0,10	-0,50	-0,15	-0,07	0,04	-0,52	-0,07	-0,32	-0,36	0,37
EC rest	-0,10	0,21	-0,17	-0,12	-0,29	-0,20	-0,14	0,20	0,00	-0,03	-0,05	0,19	-0,38	0,00	-0,57	-0,05	0,39
EO clenching	-0,11	0,18	0,06	-0,08	0,10	0,12	-0,18	-0,16	-0,36	0,32	0,01	0,21	-0,42	0,10	-0,34	-0,50	0,53
EC clenching	-0,27	0,12	0,04	-0,01	0,10	0,23	-0,10	-0,02	-0,27	0,24	-0,12	0,06	-0,44	-0,13	-0,16	-0,30	0,31
EO clenching on cotton rolls	-0,35	-0,06	-0,02	0,08	-0,08	-0,01	-0,16	0,06	-0,20	0,24	-0,31	0,16	-0,75	-0,30	-0,34	-0,29	0,15
EC clenching on cotton rolls	-0,40	-0,09	0,03	-0,23	-0,32	0,13	-0,20	0,20	-0,04	-0,21	-0,38	0,09	-0,28	0,28	-0,20	-0,16	0,45
**Mean velocity**																	
EO rest	0,08	0,04	0,28	-0,29	0,32	0,22	0,32	-0,36	-0,32	0,60	0,52	-0,13	-0,34	-0,12	-0,08	-0,35	0,10
EC rest	-0,12	-0,23	0,10	-0,23	0,15	-0,09	-0,05	-0,57	-0,31	0,01	0,16	-0,28	-0,05	0,36	-0,14	-0,17	0,09
EO clenching	0,17	0,04	0,34	-0,25	0,40	0,38	0,33	-0,40	-0,19	0,45	0,52	-0,16	-0,09	0,15	0,13	-0,16	0,02
EC clenching	-0,08	-0,23	-0,08	-0,12	0,45	0,00	0,12	-0,62	-0,34	0,19	0,29	-0,46	0,05	0,20	0,27	-0,23	-0,16
EO clenching on cotton rolls	-0,13	-0,17	0,21	-0,25	0,36	0,09	0,17	-0,61	-0,42	0,31	0,37	-0,31	-0,29	0,16	0,04	-0,32	-0,01
EC clenching on cotton rolls	-0,16	-0,21	0,04	-0,17	0,30	0,07	0,18	-0,47	-0,28	0,32	0,32	-0,26	-0,27	0,18	-0,01	-0,31	-0,02
**Foot contact area**																	
rest L	0,29	0,37	0,20	-0,01	-0,16	0,28	0,26	0,32	0,13	0,27	0,27	0,50	-0,05	-0,05	-0,10	-0,26	0,28
rest R	0,24	0,29	0,34	-0,04	0,00	0,26	0,17	0,17	0,11	0,31	0,25	0,50	-0,01	0,06	-0,08	-0,28	0,27
clenching L	0,30	0,33	0,16	0,13	-0,13	0,30	0,23	0,43	0,39	0,13	0,15	0,54	0,13	0,05	0,02	-0,02	0,10
clenching R	0,34	0,36	0,31	-0,08	-0,06	0,27	0,21	0,20	0,14	0,30	0,33	0,58	0,03	0,20	-0,11	-0,33	0,36
clenching on cotton rolls L	0,18	0,27	0,23	0,19	-0,05	0,19	0,08	0,52	0,31	0,38	0,15	0,73	-0,10	-0,13	-0,18	-0,24	0,25
clenching on cotton rolls R	0,13	0,18	0,24	-0,08	0,11	0,29	0,13	0,30	0,23	0,48	0,19	0,55	-0,01	0,13	-0,02	-0,43	0,35

AsI-index of asymmetry; DA- anterior bellies of the digastric muscles; EC – eyes closed; EO – eyes open; FCAI - Functional Clenching Activity Indice; L – left side; MM – masseter muscle; R – right side; SMC – sternocleidomastoid muscle; TA – temporalis anterior

Table S3. Correlations of static balance and posture parameters and EMG indicators in the mixed group.

Variable	Rest				Clenching				Clenching on cotton rolls				FCAI				
	AsI - TA	AsI - MM	AsI - SCM	AsI - DA	AsI - TA	AsI - MM	AsI - SCM	AsI - DA	AsI - TA	AsI - MM	AsI - SCM	AsI - DA	R	L	total	TA	MM
**Sway path length**																	
EO rest	-0,04	0,03	-0,30	0,29	0,00	-0,10	0,27	0,06	-0,48	-0,28	0,24	-0,16	0,09	-0,43	-0,24	0,07	-0,22
EC rest	-0,07	0,07	-0,19	0,47	-0,04	-0,08	0,18	-0,12	-0,47	-0,13	0,38	-0,28	0,03	-0,38	-0,26	0,00	-0,19
EO clenching	0,04	-0,14	-0,38	0,24	0,10	-0,05	0,34	0,10	-0,56	-0,30	0,16	-0,09	0,16	-0,35	-0,16	0,09	-0,36
EC clenching	0,02	0,07	-0,26	0,39	-0,04	0,09	0,18	0,12	-0,51	-0,12	0,03	-0,05	0,18	-0,27	-0,12	0,17	-0,26
EO clenching on cotton rolls	-0,03	0,11	-0,33	0,43	-0,02	-0,08	0,36	-0,02	-0,51	-0,27	0,19	-0,26	0,19	-0,50	-0,17	-0,03	-0,24
EC clenching on cotton rolls	0,27	0,21	-0,13	0,44	-0,09	-0,03	0,25	-0,04	-0,44	-0,01	0,18	-0,09	0,39	-0,12	0,11	0,09	-0,15
**Ellipse area**																	
EO rest	-0,12	-0,12	-0,49	0,08	0,01	-0,21	0,30	-0,33	-0,81	-0,14	0,03	-0,26	-0,15	-0,51	-0,26	-0,52	-0,51
EC rest	-0,05	0,04	-0,47	-0,19	0,12	-0,12	0,35	-0,21	-0,37	-0,17	0,00	-0,36	0,06	-0,44	-0,06	-0,49	-0,10
EO clenching	0,08	-0,09	-0,47	0,19	0,26	0,04	0,39	-0,08	-0,71	-0,15	-0,21	-0,02	0,14	-0,34	0,02	-0,21	-0,49
EC clenching	-0,14	0,03	-0,33	-0,13	-0,05	-0,06	0,25	-0,14	-0,57	-0,08	-0,04	-0,25	0,12	-0,35	0,03	-0,67	-0,09
EO clenching on cotton rolls	-0,16	-0,10	-0,55	0,04	0,11	-0,12	0,26	-0,39	-0,75	-0,09	-0,12	-0,34	-0,15	-0,49	-0,25	-0,51	-0,47
EC clenching on cotton rolls	-0,33	0,06	-0,41	-0,03	-0,16	-0,17	0,19	-0,17	-0,60	-0,13	-0,13	-0,35	0,08	-0,49	-0,07	-0,72	-0,25
**Mean velocity**																	
EO rest	-0,08	-0,01	-0,37	0,29	0,07	-0,12	0,31	0,07	-0,39	-0,34	0,27	-0,13	0,04	-0,39	-0,28	0,14	-0,24
EC rest	-0,05	-0,02	-0,29	0,46	0,03	-0,10	0,23	-0,15	-0,47	-0,13	0,44	-0,30	-0,09	-0,42	-0,37	-0,02	-0,28
EO clenching	0,14	-0,10	-0,37	0,32	0,05	-0,09	0,34	0,02	-0,53	-0,24	0,24	-0,11	0,20	-0,31	-0,13	0,11	-0,39
EC clenching	0,06	-0,06	-0,36	0,30	0,00	0,06	0,22	0,13	-0,63	-0,13	0,12	-0,06	-0,02	-0,49	-0,32	-0,01	-0,38
EO clenching on cotton rolls	-0,01	0,03	-0,26	0,40	-0,02	-0,09	0,47	0,04	-0,54	-0,24	0,23	-0,23	0,26	-0,39	-0,06	-0,10	-0,27
EC clenching on cotton rolls	0,29	0,11	-0,24	0,39	0,00	0,14	0,24	0,01	-0,54	0,04	0,14	-0,08	0,38	-0,16	0,11	-0,02	-0,21
**Foot contact area**																	
rest L	-0,17	-0,23	0,28	-0,09	-0,14	-0,20	-0,02	0,04	0,35	0,01	0,01	0,26	0,06	0,50	0,29	0,07	-0,16
rest R	-0,42	-0,18	0,30	-0,10	-0,16	-0,41	-0,08	0,03	0,31	-0,29	0,07	0,17	0,01	0,30	0,16	0,06	-0,06
clenching L	-0,27	-0,29	0,18	0,04	-0,03	-0,16	0,12	0,07	0,31	-0,07	0,03	0,18	-0,03	0,31	0,15	0,09	-0,28
clenching R	-0,44	-0,24	0,17	-0,08	-0,07	-0,37	0,04	0,00	0,34	-0,32	0,10	0,17	-0,07	0,26	0,08	0,10	-0,18
clenching on cotton rolls L	-0,21	-0,36	0,18	-0,08	-0,02	-0,18	0,08	0,04	0,39	-0,03	0,08	0,16	-0,02	0,43	0,22	0,09	-0,22
clenching on cotton rolls R	-0,19	-0,22	0,39	0,00	-0,12	-0,39	0,04	0,16	0,41	-0,26	0,05	0,33	0,08	0,38	0,26	0,26	-0,16

AsI-index of asymmetry; DA- anterior bellies of the digastric muscles; EC – eyes closed; EO – eyes open; FCAI - Functional Clenching Activity Indice; L – left side; MM – masseter muscle; R – right side; SMC – sternocleidomastoid muscle; TA – temporalis anterior

Table S4. Correlations of static balance and posture parameters and EMG indicators in the articular group.

Variable	Rest				Clenching				Clenching on cotton rolls				FCAI				
	AsI - TA	AsI - MM	AsI - SCM	AsI - DA	AsI - TA	AsI - MM	AsI - SCM	AsI - DA	AsI - TA	AsI - MM	AsI - SCM	AsI - DA	R	L	total	TA	MM
**Sway path length**																	
EO rest	0,17	-0,28	0,39	-0,01	0,02	0,07	0,04	0,14	-0,09	0,33	-0,17	0,14	-0,12	0,00	0,03	0,11	0,04
EC rest	0,02	-0,35	0,06	0,11	-0,27	-0,11	0,12	-0,21	0,01	-0,03	-0,08	0,19	0,07	0,08	0,07	-0,13	0,21
EO clenching	0,13	-0,36	0,22	0,06	-0,07	0,09	0,02	0,12	-0,12	0,15	-0,15	0,16	-0,06	0,11	0,19	-0,06	0,10
EC clenching	0,29	-0,30	0,17	0,06	-0,14	0,04	0,04	-0,14	0,16	0,19	-0,04	0,16	0,01	-0,01	0,12	-0,06	0,27
EO clenching on cotton rolls	0,19	-0,32	0,27	-0,01	-0,13	-0,08	0,03	0,11	-0,16	0,21	-0,13	0,23	-0,14	-0,01	0,07	-0,04	-0,06
EC clenching on cotton rolls	0,05	-0,52	0,14	0,12	-0,24	0,01	-0,05	-0,08	0,05	0,05	-0,25	0,26	0,16	0,19	0,32	-0,19	0,20
**Ellipse area**																	
EO rest	-0,24	0,25	-0,43	-0,36	-0,15	-0,40	0,05	-0,08	-0,23	-0,25	0,08	0,13	0,22	0,00	-0,14	-0,06	0,03
EC rest	0,33	0,15	-0,21	-0,48	0,07	-0,07	0,07	-0,25	-0,43	0,12	0,10	-0,11	-0,11	0,00	-0,18	0,27	-0,15
EO clenching	-0,18	0,32	-0,49	-0,57	0,25	-0,13	-0,14	-0,02	-0,22	-0,22	-0,22	0,22	0,21	-0,03	-0,11	0,00	0,09
EC clenching	0,24	0,19	-0,23	-0,50	0,24	0,03	0,15	-0,34	-0,40	0,07	0,07	-0,11	-0,26	-0,20	-0,32	0,23	-0,22
EO clenching on cotton rolls	-0,16	0,30	-0,35	-0,41	0,18	-0,19	-0,19	0,00	-0,25	-0,14	-0,21	0,15	0,27	0,13	-0,04	0,14	0,05
EC clenching on cotton rolls	0,03	-0,08	-0,26	-0,23	0,03	0,01	0,06	-0,46	-0,44	0,01	0,09	-0,24	-0,07	0,18	0,00	0,14	-0,39
**Mean velocity**																	
EO rest	0,17	-0,28	0,39	-0,01	0,02	0,07	0,04	0,14	-0,09	0,33	-0,17	0,14	-0,12	0,00	0,03	0,11	0,04
EC rest	0,03	-0,37	0,09	0,14	-0,25	-0,09	0,14	-0,24	0,03	-0,01	-0,06	0,15	0,05	0,07	0,07	-0,12	0,20
EO clenching	0,09	-0,53	0,13	-0,07	-0,19	0,09	0,19	-0,08	-0,09	0,23	0,01	0,04	0,02	0,06	0,19	-0,13	0,02
EC clenching	0,24	-0,37	0,14	0,05	-0,18	-0,01	0,15	-0,19	0,18	0,15	0,10	0,14	-0,09	-0,06	0,06	-0,23	0,18
EO clenching on cotton rolls	0,21	-0,27	0,21	-0,18	-0,03	0,00	0,12	0,08	-0,29	0,30	-0,02	0,05	-0,19	-0,04	0,00	0,04	-0,17
EC clenching on cotton rolls	0,10	-0,48	0,38	0,15	-0,04	0,07	0,10	-0,11	0,22	0,13	-0,15	0,20	-0,06	-0,09	0,11	-0,17	0,17
**Foot contact area**																	
rest L	-0,22	-0,14	-0,01	0,28	0,26	0,37	-0,04	-0,13	0,22	0,08	-0,07	-0,37	0,40	0,16	0,35	0,35	-0,11
rest R	-0,21	-0,21	0,11	0,21	0,34	0,46	0,06	-0,18	0,43	0,19	0,02	-0,39	0,33	0,02	0,29	0,24	-0,04
clenching L	-0,15	-0,11	0,04	0,20	0,35	0,43	-0,01	-0,13	0,24	0,15	-0,06	-0,40	0,35	0,12	0,29	0,40	-0,07
clenching R	-0,29	-0,10	0,01	0,12	0,34	0,41	0,08	-0,18	0,33	0,13	0,02	-0,39	0,36	0,09	0,21	0,28	0,03
clenching on cotton rolls L	-0,14	0,09	-0,12	0,02	0,40	0,19	-0,07	-0,24	0,04	-0,01	0,00	-0,37	0,29	0,13	0,17	0,36	-0,20
clenching on cotton rolls R	-0,26	-0,01	-0,14	0,04	0,44	0,29	0,06	-0,14	0,28	-0,06	0,02	-0,33	0,41	0,13	0,22	0,28	0,01

AsI-index of asymmetry; DA- anterior bellies of the digastric muscles; EC – eyes closed; EO – eyes open; FCAI - Functional Clenching Activity Indice; L – left side; MM – masseter muscle; R – right side; SMC – sternocleidomastoid muscle; TA – temporalis anterior
